# Supplementary figures and images for: Findings from the Tushirikiane-4-MH (supporting each other for mental health) mobile health–supported virtual reality randomized controlled trial among urban refugee youth in Kampala, Uganda
Source: Glob Ment Health (Camb). 2025 Jan 23;12:e12. doi: 10.1017/gmh.2025.3 (PMC11810762; doi:10.1017/gmh.2025.3)

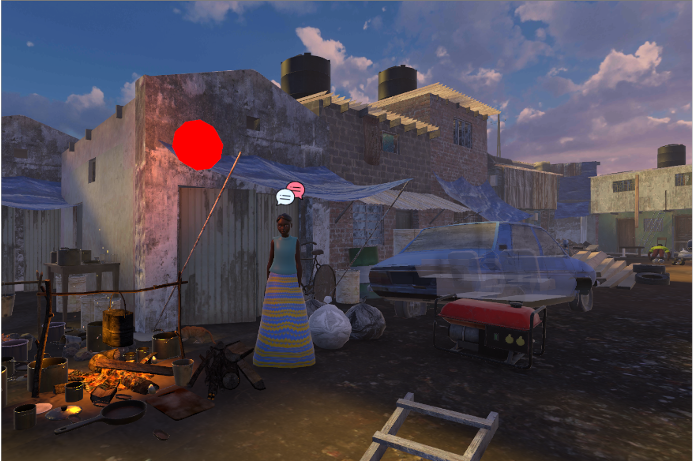

Supplement: Logie et al. supplementary material [file S2054425125000032sup001.zip › Untitled.tiff]

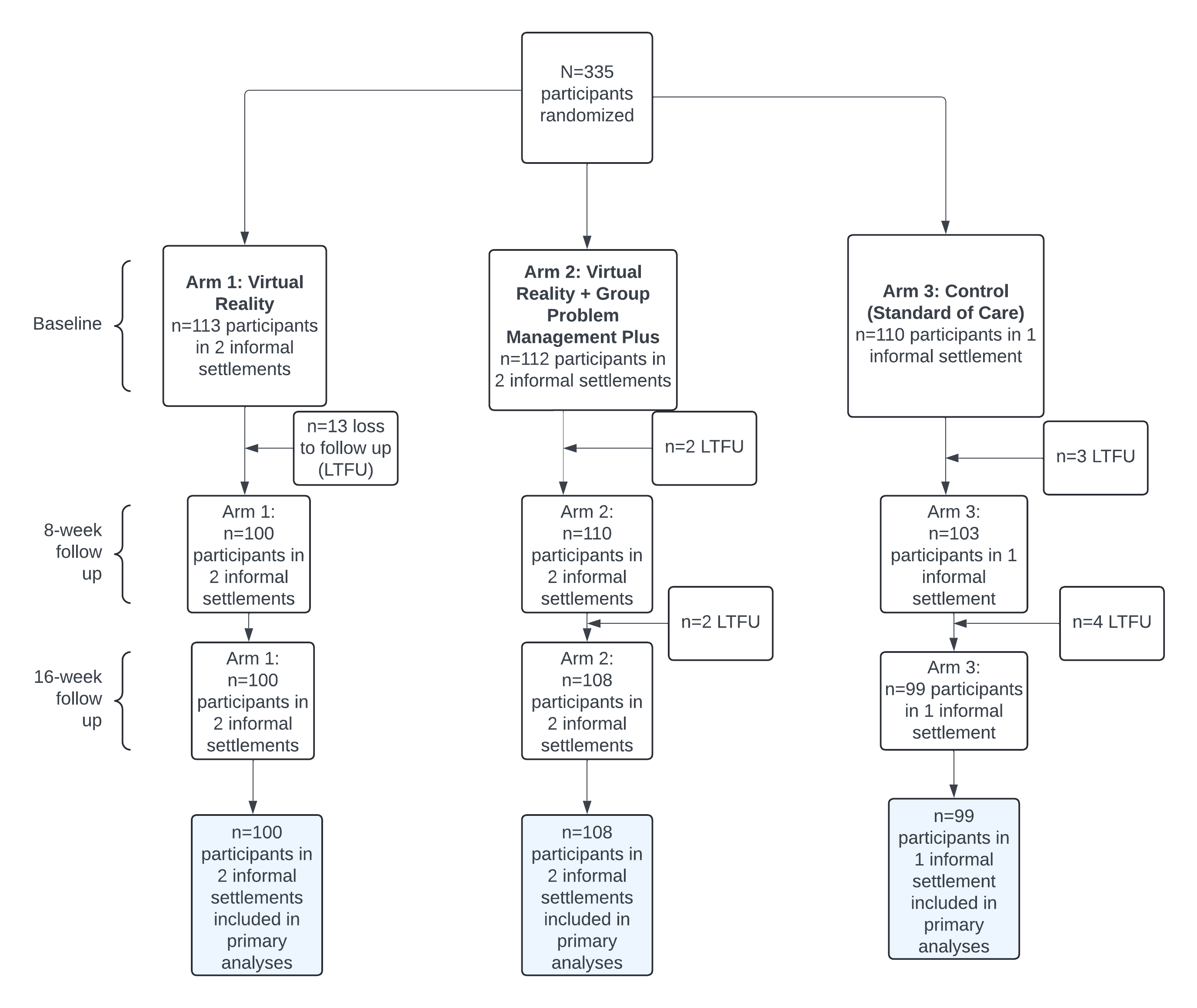

Supplement: Logie et al. supplementary material [file S2054425125000032sup001.zip › VR study flow.png]
